# STAMP alters the growth of transformed and ovarian cancer cells

**DOI:** 10.1186/1471-2407-10-128

**Published:** 2010-04-07

**Authors:** Yuanzheng He, John A Blackford, Elise C Kohn, S Stoney Simons

**Affiliations:** 1 Steroid Hormones Section, Clinical Endocrinology Branch, NIDDK, National Institutes of Health, Bethesda, MD 20892-1772, USA; 2 Lab of Structural Sciences, Van Andel Institute, 333 Bostwick Avenue, Grand Rapids, MI 49503, USA; 3 Molecular Signaling Section, Medical Oncology Branch, NCI, National Institutes of Health, Bethesda, MD 20892, USA

## Abstract

**Background:**

Steroid receptors play major roles in the development, differentiation, and homeostasis of normal and malignant tissue. STAMP is a novel coregulator that not only enhances the ability of p160 coactivator family members TIF2 and SRC-1 to increase gene induction by many of the classical steroid receptors but also modulates the potency (or EC_50_) of agonists and the partial agonist activity of antisteroids. These modulatory activities of STAMP are not limited to gene induction but are also observed for receptor-mediated gene repression. However, a physiological role for STAMP remains unclear.

**Methods:**

The growth rate of HEK293 cells stably transfected with STAMP plasmid and overexpressing STAMP protein is found to be decreased. We therefore asked whether different STAMP levels might also contribute to the abnormal growth rates of cancer cells. Panels of different stage human cancers were screened for altered levels of STAMP mRNA. Those cancers with the greatest apparent changes in STAMP mRNA were pursued in cultured cancer cell lines.

**Results:**

Higher levels of STAMP are shown to have the physiologically relevant function of reducing the growth of HEK293 cells but, unexpectedly, in a steroid-independent manner. STAMP expression was examined in eight human cancer panels. More extensive studies of ovarian cancers suggested the presence of higher levels of STAMP mRNA. Lowering STAMP mRNA levels with siRNAs alters the proliferation of several ovarian cancer tissue culture lines in a cell line-specific manner. This cell line-specific effect of STAMP is not unique and is also seen for the conventional effects of STAMP on glucocorticoid receptor-regulated gene transactivation.

**Conclusions:**

This study indicates that a physiological function of STAMP in several settings is to modify cell growth rates in a manner that can be independent of steroid hormones. Studies with eleven tissue culture cell lines of ovarian cancer revealed a cell line-dependent effect of reduced STAMP mRNA on cell growth rates. This cell-line dependency is also seen for STAMP effects on glucocorticoid receptor-mediated transactivation. These preliminary findings suggest that further studies of STAMP in ovarian cancer may yield insight into ovarian cancer proliferation and may be useful in the development of biomarker panels.

## Background

Cell proliferation is a complicated and poorly understood process with contributions from many factors. Consequently identifying the causes for abnormal cell propagation, such as in cancers, has been an even more daunting task. Recent evidence suggests that mutant pathways (produced by a variety of mutant or dysregulated proteins) as opposed to a single mutant or dysregulated protein are responsible for at least some cancers [1]. One pathway that is well known for its role with normal cell growth and differentiation involves the classical steroid receptors. Androgen (ARs), estrogen (ERs), and progesterone receptors (PRs) are best recognized for their contribution in the morphogenesis of reproductive tissues [2-4]. Conversely, preventing the actions of ARs and ERs has been the major treatment of choice for many years in combating hormone-sensitive prostate and breast cancers respectively [5,6]. Glucocorticoids are clinically invaluable in treating lymphoid cancers and attenuating immune responses [7] while mineralocorticoid agonists are now thought to have significant effects on the heart and vasculature [8,9]. A major component in the expression of steroid receptor-regulated gene expression is coactivators and corepressors, which typically elevate or inhibit the action of receptor-agonist complexes [10-14].

STAMP is a novel coregulator that enhances the ability of p160 coactivator family members TIF2 and SRC-1 to augment gene induction by AR, glucocorticoid receptor (GR), and PR. STAMP also modulates the potency (or EC_50_) of agonists and the partial agonist activity of antisteroids [15,16]. These modulatory activities of STAMP are not limited to gene induction but are also observed for GR-mediated gene repression [17]. These actions appear to proceed via the binding of internal and C-terminal regions of STAMP to coactivators and receptors respectively in a manner that includes steroid-mediated recruitment of STAMP to the enhancer region of regulated genes. Interestingly, STAMP does not interact with ER or members of the nuclear receptor sub-family such as thyroid receptor, PPAR, RAR, or RXR [15].

The physiological role of STAMP, however, remains unclear. The absence of STAMP paralogs in the human genome suggests an important function. STAMP has been included as a member of the tyrosine tubulin ligase-like (TTLL) family [18] on the basis of containing a tyrosine tubulin ligase (TTL) domain. However, this grouping is not applicable to several of the biological function assays of STAMP in that the coregulatory activity of STAMP with GRs does not require the TTL domain [15]. Differential detection in Northern blots [15] and qPCR analysis of mouse tissues (G-S Lee and SS Simons, unpublished) is consistent with a role for STAMP in brain, ovaries, and testes. In addition, the importance of steroid receptors in normal and aberrant cell growth combined with STAMP binding to, and modulating the activity of, AR, GR, and PR suggests a possible role for STAMP in regulating the growth of some cells and cancers.

Ovarian cancer is the fifth most common cancer in the United States and is responsible for 4% of new cancer cases, and 5% of the cancer deaths, occurring in women each year [19,20]. The major cause of death is from epithelial ovarian tumors, which constitute the large majority of ovarian cancers [21]. Ovarian cancer in general has a 5-year survival rate of over 90% if diagnosed and treated early, when the cancer is confined to the ovaries. Unfortunately, due to ovarian cancer's non-specific symptoms and the lack of reliable early detection methods, only about 20% of all cases are found at this beginning stage. If caught in an advanced stage, the 5-year survival rate can be as low as 29% [22,23]. The inability to identify and treat this cancer early underscores the necessity for better understanding its biology and unique gene and protein expression panels. Unfortunately, the heterogeneity of epithelial ovarian cancers complicates this task. It has recently been suggested that ovarian cancer, like breast cancer, may be subdivided by differences in gene expression panels [24,25]. Therefore, it is likely that the mechanism(s) of ovarian cancer growth and metastasis will involve multiple biochemical pathways, as has been concluded for pancreatic and breast cancers [1,26].

We now report that STAMP has the physiologically relevant effect of influencing the growth rate of some cells in a manner that appears independent of steroid hormones. Of potential clinical interest is our finding that STAMP mRNA levels are elevated in human ovarian cancer samples (stages I-IV), although the effects of STAMP levels on ovarian cancer cell growth vary. We suggest that the effects of STAMP are cell-line dependent and further investigation into this unique finding may advance our understanding of tumorigenesis and progression of some human ovarian cancers.

## Methods

### Cell lines and reagents

Various epithelial ovarian cancer cell lines were obtained as gifts: SHIN3 and OVCAR5 from Mark Levine (NIDDK), and A1847, A2780, IGROV1, OVCAR10, PEO-1, and 2008 from Charles Drescher and Beatrice Knudsen (Fred Hutchinson Cancer Research Center, Seattle WA). Renilla TS was a gift from Nasreldin M. Ibrahim, Otto Fröhlich, and S. Russ Price (Emory University School of Medicine). STAMP SMARTpool siRNA was purchased from Dharmacon (#M-015406), Lamin A/C siRNA from Qiagen (#1027320), STAMP Taqman gene expression assay probe (#Hs00367300_m1) and human GAPDH control probe (#4310884E) from ABI, and siLentFect siRNA transfection reagent from Bio-Rad #170-3361. GR plasmid (pSVLGR) is a gift from Keith Yamamoto (Univ. of Cal., SF). The CellTiter 96 AQueous One Solution cell growth assay is supplied by Promega. Mouse monoclonal anti-TIF2 antibody is from Bethyl Labs (#A300-345A). Rabbit polyclonal anti-α-tubulin antibody (used at 15 ng/ml) is from Abcam (#ab4074). The following primers were used for qRT-PCR of both human and rat GR (numbers in parentheses are positions in human GR cDNA): GTCTTCAGGCTGGAATGAACC (1443-1462) and CTTTGCCCATTTCACTGCTGC (1737-1717).

### Stably transfected 293 cells

HEK293 cells were always grown in high glucose DMEM (Invitrogen) with 10% fetal bovine serum, sodium pyruvate (Cellgro), and MEM nonessential amino acids (Cellgro) to 70% confluency in 100 mm dishes. Cells were transfected with 10 μg of FLAG-STAMP/pCDNA3.1neo or pCDNA3.1neo using 23 μl Fugene 6 (Roche Diagnostics, Branford, CT) for 24 hr. The media was changed and the cells grown for another 24 hr before being washed (PBS without Ca^++^/Mg^++^; Invitrogen), trypsinized, diluted 1:50-1000 in media containing 1 mg/ml G418 (Invitrogen), plated in 100 mm dishes and grown for 14 days. Colonies were selected and expanded first in 24 well plates for 10 days and then in 6 well plates until there were enough cells, all with 0.5 mg/ml G418. Positive clones were tested for Flag-STAMP expression by Western blotting with anti-STAMP antibody prepared by Covance (3^rd ^bleed, HL5761). All HEK293 stable cells used for experiments were grown in media free of G418 for at least 2 weeks unless otherwise noted.

### Cell growth assay

Cells were washed (PBS without Ca^++^/Mg^++)^, trypsinized, and then seeded at 10,000 cells/well in 96 well culture plates. Cell growth at the indicated points (day of seeding = Day 0) was determined by adding 20 μl of CellTiter One Solution/well and measuring the OD492 3 hours later in a Beckman Count DTX880 as recommended by the supplier (Promega). Medium without cells was used as the blank control.

### FACS and cell counting

Cells (grown with 0.1 mg/ml G418) were washed 3 times with PBS without calcium or magnesium (Invitrogen), counted with a Beckman Coulter Vi-Cell XR2.02, and seeded at 50,000 viable cells/well in 6 well dishes in 3 ml of medium without G418. After 24 hours, the cells were washed once with PBS, trypsinized, resuspended in 500 μl of medium, and placed on ice. Cells were incubated at 0°C with 1 μl of 5 mg/ml of propidium iodide (Invitrogen) immediately prior to analysis on a Becton Dickinson FacsCalibur flow cytometry system and counting with a Beckman Coulter Vi-Cell XR2.02.

### Tissue scan panels for endogenous STAMP determination

Tissue Scan panels (8 common cancers [#CSRT101], kidney cancer [#HKRT101], ovarian cancer [#HORT101 and HORT102], and breast cancer [#BCRT101]) and the accompanying pathology and tumor staging reports are available from OriGene (Rockville, MD). Each panel contains pre-normalized cDNA arrays prepared from pathologist-verified tumor tissues. Each well of the panel has same amount total cDNA. Thus, any target gene (e.g. STAMP) variation in different wells reflects real changes between tumor samples. Quantitative real-time PCR (qRT-PCR) was run once for each panel (plate) using validated STAMP primers according to manufacturer's instructions. To determine the absolute level of STAMP, 10 μl 2× Taqman PCR master, 1 μl STAMP Taqman probe, and 9 μl water (total 20 μl) was added to each well of the 96 well plate, as recommended by OriGene. Then real-time qRT-PCR was run on an ABI HT 7900 according to the manufacturer's instructions.

### Transient transfections of stably transfected cells withsiRNAs or plasmids

For 6 well plates, cells were seeded at 5000 per well one day before transfection. One hour before transfection, 1.25 ml of fresh medium was added. siRNA (75 pM) was incubated for 20 min at r.t. with 1 μl siLentFect reagent in Opti-mem medium (total of 250 μl) before being added to Opti-mem medium in each well (final concentration of siRNA = 50 nM). For 96 well plates, cells were seeded at 500-2000 per well and 50 μl of fresh medium was added one hour before transfection. Then 3.5 pM of siRNA was mixed with 0.05 μl siLentFect in a total of 20 μl of Opti-mem medium for 20 min before being added to each well. One day later, fresh medium was added to each well in both protocols.

Total RNA isolation, reverse transcription, real-time PCR, and luciferase assays of transiently transfected GREtkLUC were performed as previously described [15]. For transient transfection experiments with GREtkLUC, VA (1 × 10^4^) or S13 (2 × 10^4^) cells in 300 μl of media were transfected for 24 hr using 0.8 μl/well of Fugene 6 and either 0, 10 or 30 ng pSVLGR plus 100 ng GREtkLuc and 10 ng Renilla TS. Total transfected DNA was maintained at 300 ng/well with PBSK+. The cells were then induced with steroids in fresh media for 24 hr, separated from the media, and lysed for 20 min with 300 μl of passive lysis buffer (Promega) at room temperature on a rotating shaker. Lysate (50 μl) was then loaded into 96 well luminometer plates and read in a Berthold luminometer.

### Statistical analysis

Unless otherwise noted, the values of *n *independent experiments, performed in triplicate for Luciferase assays, were analyzed for statistical significance by the two-tailed Student's t test using InStat 2.03 (GraphPad Software, San Diego, CA). In every case, each average of triplicates was treated as one value of the n experiments. When the difference between the SDs of two populations was significantly different, the Mann-Whitney or Alternate Welch t test was used. A nonparametric test was used if the distribution of values was non-Gaussian. Best-fit curves (R^2 ^almost always ≥ 0.95) following Michaelis-Menten kinetics were obtained for the dose-response experiments with KaleidaGraph (Synergy Software, Reading, PA).

## Results

### STAMP overexpression decreases growth of human embryonic kidney 293 cells

**Figure 1:**
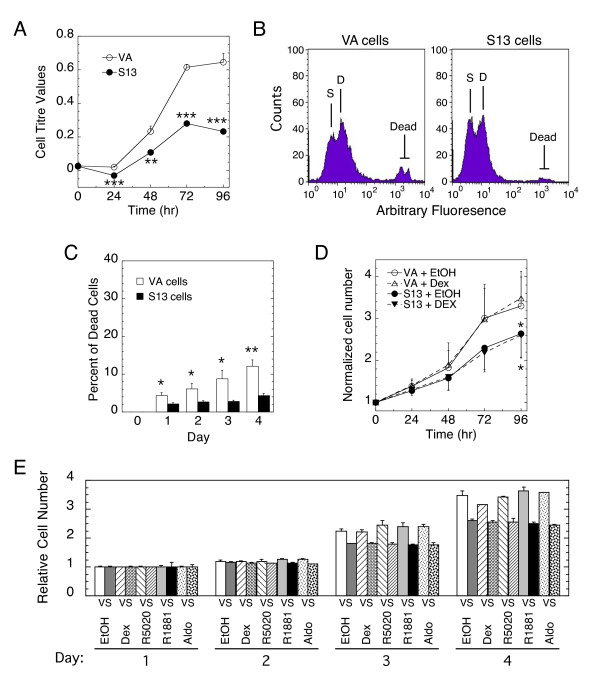
**Effect of stably transfected STAMP on the growth rate of 293 cells**. (A) Increased STAMP decreases cell growth. The number of cells in replicate plates of clone S13 (S) and VA (V) cells (no steroid, cells removed from G418 1 day prior) after increasing days was assessed by the CellTiter One Solution assay. The average value (± S.E.M.) of four independent experiments is shown. **P < 0.005, ***P < 0.0005. (B&C) FACS analysis of VA and S13 cells (no steroid, cells removed from G418 upon seeding; S = single cells, D = double cells). The number of dead cells after increasing days in one of three representative experiments is plotted in C (± SD, n = 3, *P < 0.05, **P < 0.005). (D) Growth of VA and S13 cells after removal from G418 for more than 1 month. Experiment was conducted and plotted (± S.E.M., n = 4, *P < 0.05) as in A except that 1 μM Dex was added to cells as indicated. (E) Increased STAMP decreases cell growth ± steroid. Experiment was conducted as in D except that four different steroids (100 nM aldosterone, 1 μM Dex, 20 nM R5020, and 10 nM R1881) were added. Similar results were obtained in two additional independent experiments. In some cases (e.g., S13 cells in Fig. 1A), the error bar length is smaller than the symbol.

Two colonies of 293 cells containing stably transfected STAMP were selected for study. Both colonies gave less than 50% of the increase of control cells with stably transfected empty vector (clone VA) after 3-4 days, as determined by counting of isolated cells (43 ± 19% [S.D., n = 9] and 33 ± 7% [S.D., n = 2] for clones 13 and 10 respectively vs. VA cells). One colony (clone S13) was selected for further study. When removed from G418 selection and immediately examined by "Cell Titre" assay, clone S13 cells show a 65% reduction in cell replication (Fig. 1A). Automated cell counting gave similar results (data not shown). FACS analysis showed no increase in the proportion of dead cells in S13 vs. VA cells (Figs. 1B&C). Thus, the lower density of S13 cells over time is due to slower growth as opposed to increased cell death.

Given the undetectable level of STAMP in VA cells by Western blotting (data not shown), qRT-PCR was used to determine the relative amounts of STAMP. STAMP mRNA in clone S13 was 32-fold higher than in VA cells but decreased by a factor of two to 14-fold after withdrawal from G418 for a least 3 months. As expected, these reduced levels of excess STAMP were reflected in a smaller reduction of cell growth, with or without glucocorticoid (Fig. 1D). The growth rate of clone S13 as a percent of clone VA cells was the same with EtOH (77 ± 0.02%; S.E.M, n = 10, P < 0.0001) and Dex (76 ± 0.03%; S.E.M., n = 9, P < 0.0002).

In view of the ability of STAMP to also alter the induction properties of PR and AR [15,16], we asked if the addition of any other steroids could influence the growth of clone S13 vs. control (clone VA) cells. As shown in Fig. 1E, saturating levels for receptor binding of progestin (R5020), androgen (R1881), or mineralocorticoid (aldosterone) also did not change the growth rate of cells with or without overexpressed STAMP.

### STAMP levels in human tumor samples

**Figure 2:**
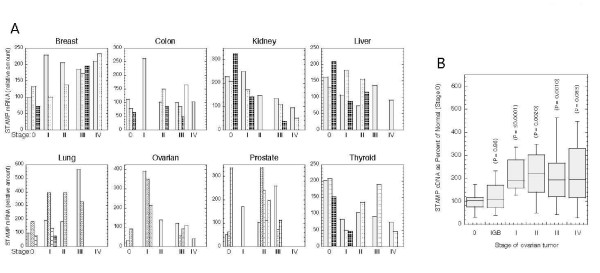
**Levels of STAMP mRNA in different human tumor samples**. (A) Survey of eight human tumor types. Levels of STAMP mRNA in individual samples of OriGene Tissue Scan panel (each bar corresponds to a single tissue sample) were determined as described in Materials and Methods and plotted (relative to the STAMP mRNA level of the first sample in the Breast panel) vs. tumor stage. (B) STAMP mRNA in human ovarian cancers. Levels of STAMP mRNA in two different large scale OriGene Tissue Scan panels of only ovarian cancers was determined as in A. The data from both panels were combined and plotted (box plot) as percent of the average STAMP level in normal (Stage 0) samples (n = 13) for the different stages (n for Stage IGB [low malignant potential] = 3, Stage I = 12, Stage II = 12, Stage III = 34, Stage IV = 9). P values were determined for ΔCt values from the qRT-PCR assay before conversion to percent of STAMP in normal samples, which was plotted.

In view of the effect of STAMP on 293 cell growth, we asked if the abnormal growth of any cancers might be influenced by the levels of endogenous STAMP. Standardized amounts of cDNAs from each stage of 8 common human cancers were obtained from OriGene. Quantitative real time PCR analysis with STAMP TaqMan probes was performed to determine the amount of STAMP mRNA in each tumor sample (Additional file 1: Table S1). As shown in Fig. 2A where the results from individual samples are plotted, this preliminary screen suggested that STAMP levels in kidney cancers gradually decrease as the tumor stage progresses while STAMP levels might be elevated in Stage I of ovarian cancers and several stages of breast cancer.

These pilot-study observations were pursued by analyzing larger panels containing 48 samples from various stages of these three cancers. This more extensive study displayed no significant differences in STAMP levels among various kidney and breast cancer samples (data not shown). However, data from a total of 83 additional samples in two other ovarian cancer panels revealed a statistically significant, two-fold elevation of STAMP mRNA in tumors of stages I-III relative to "normal" ovarian tissue (n = 13) (Fig. 2B; see Additional file 2: Table S2 for description and grading of samples plus the raw qPCR data). We recognize that this result may be misleading as the "normal" is most likely enriched for normal ovarian stroma, which is nearly devoid of epithelial cells that are found primarily on the capsular surface of the ovary. The difference between overt Stage I-III cancers and the three low malignant potential, borderline tumors (IGB) is significant (P = 0.033 by one-tailed Student t-test) because low malignant potential ovarian neoplasm is a nonmalignant but epithelial process. These two findings led us to further investigate the role of STAMP in ovarian cancer.

Nine cultured ovarian cancer cell lines were examined: Hey A8, SKOV3, OVCAR8, A1847, PEO-1, OVCAR10, A2780, 2008, and IGROV1. The STAMP levels in three randomly selected, untreated cell lines (Hey A8, SKOV3, OVCAR8) were determined and normalized to the amount in Hey A8 cells, which was arbitrarily selected as a reference value. STAMP mRNA levels in these ovarian cancer cell lines do not appear abnormal. They are slightly higher than that in two other transformed cell lines (U2OS.rGR osteosarcoma cells with stably transfected GR and the control 293 VA cells) and much less than in the clone S13 293 cells with stably transfected STAMP (Table 1).

**Table 1:** Relative STAMP mRNA levels in ovarian cancer tissue culture cells

Cells:	Hey A8	SKOV 3	OVCAR 8	U2OS.rGR	293 clone VA	293 clone S13
Level:	100	269 ± 67	113 ± 8	59 ± 6	149	1864

STAMP mRNA levels in the indicated cells were determined by qRT-PCR as described in Materials and Methods and then normalized to that seen in HeyA8 cells (which was arbitrarily selected as reference). Values are the averages ± S.E.M. of 3 experiments (one experiment for 293 cells).

### Role of STAMP in proliferation of ovarian cancer cells

**Figure 3:**
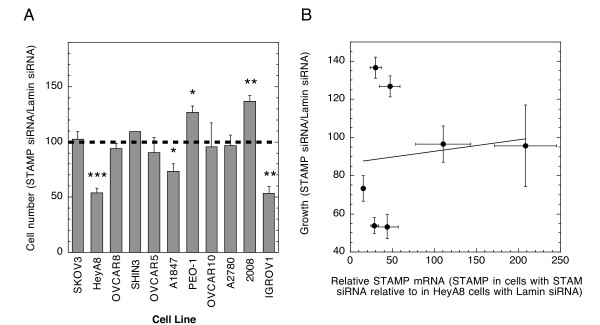
**Effect of STAMP siRNA on growth of ovarian cancer tissue culture cells**. (A) Growth of ovarian tissue culture cells treated with STAMP siRNA relative to Lamin siRNA. The growth of cells 4 days after being transfected with STAMP siRNA or Lamin siRNA was determined as in Fig. 1. The average data ± S.E.M. for 2-6 determinations (n = 12 for HeyA8 cells) are plotted as growth of cells treated with STAMP siRNA as percent of growth of cells treated with Lamin siRNA. * P < 0.05, ** P < 0.005, *** P < 0.0005. (B) Growth rate vs. STAMP mRNA levels. The relative growth rate (STAMP siRNA vs. Lamin siRNA) of each of the cell lines in A (± S.E.M.) was plotted against the level of STAMP mRNA in the same cells after siRNA treatment relative to HeyA8 cells (± S.E.M.; length of bar may be less than symbol) given in Table 2. Straight line is the best fit by Kaleidagraph (R^2 ^= 0.016).

Because elevated STAMP decreases the growth rate of 293 cells (Fig. 1), we asked if lowering the levels of endogenous STAMP with transfected STAMP siRNA would increase the growth rate of the ovarian cancer cells. After transfecting the cells with either Lamin siRNA, as a control, or a SMARTpool (Dharmacon) of STAMP siRNAs, we again determined the STAMP levels. On average, STAMP siRNA reduced the level of endogenous STAMP mRNA in each cell line to 28 ± 8% (S.D., n = 7 cell lines, each being the average of three independent experiments) of that seen in the same cells treated with the Lamin siRNA control (Table 2; "percent remaining"). The "absolute" values of STAMP mRNA in each cell line following STAMP siRNA treatment, after adjusting for the different initial levels in each cell line, are also given Table 2 (values are expressed relative to Hey A8 reference cells, with STAMP levels in Hey A8 cells treated with Lamin siRNA = 100). Unexpectedly, a reduced amount of STAMP mRNA now inhibits the growth of Hey A8, A1847 and IGROV1 cells, in each case compared to the growth of the same cells treated with Lamin siRNA (Fig. 3A). However, lower STAMP mRNA levels have the opposite (and initially predicted) effect on the proliferation of PEO-1 and 2008 cells, which are significantly increased. Finally, reduced STAMP mRNA levels cause no change in the propagation of SKOV3, OVCAR8, OVCAR10, A2780 and two other ovarian cancer cell lines (SHIN3 and OVCAR5) (Fig. 3A).

**Table 2:** Relative STAMP mRNA levels in ovarian cancer tissue culture cells after siRNA treatment

Cells:	Hey A8	A1847	PEO-1	OVCAR10	A2780	2008	IGROV1
Percent remaining:	29 ± 5	13 ± 3	33 ± 6	25 ± 5	33 ± 9	23 ± 3	38 ± 4
Absolute STAMP mRNA relative to HeyA8):	29 ± 5	16 ± 2	47 ± 12	208 ± 37	110 ± 32	30 ± 6	43 ± 14

STAMP mRNA levels in the indicated cells with transfected siRNA were determined by qRT-PCR as described in Materials and Methods and then normalized to that seen in HeyA8 cells (which was arbitrarily selected as reference) after transient transfection with Lamin siRNA. The data show both the percent remaining STAMP mRNA after siRNA treatment (i.e., 100 × STAMP siRNA/Lamin siRNA) and the "absolute" levels relative to STAMP in HeyA8 cells treated with Lamin siRNA. For this normalization to STAMP in Lamin siRNA-treated HeyA8 cells, the ratio (×100) of (STAMP mRNA in each STAMP siRNA-treated cell)/(STAMP mRNA in STAMP siRNA-treated HeyA8 cells) is multiplied by the ratio of (STAMP mRNA in STAMP siRNA-treated HeyA8 cells)/(STAMP in Lamin siRNA treated HeyA8 cells) in the same experiment (values ranged from 0.22 to 0.38). Values are the averages ± S.E.M. of 3 experiments.

In view of the different growth responses to lowered STAMP levels in the ovarian cancer cell lines, it seemed possible that the absolute level of STAMP, as opposed to the relative amount, might be a critical determinant for growth rates. In this case, cells with high levels of STAMP mRNA after siRNA treatment, relative to HeyA8 (see "absolute" values in Table 2), might have different growth rates than those with much lower levels. However, PEO-1 and IGROV1 cells have similar levels of STAMP mRNA after STAMP siRNA treatment ("absolute" in Table 2) but very different growth rates (Fig. 3A). The growth response of the cells treated with STAMP siRNA (Table 2) are plotted in Fig. 3B. This clearly shows that there is no correlation between the amount of STAMP mRNA in STAMP siRNA-treated cells and the growth rate of STAMP siRNA-treated cells as a percent of Lamin siRNA-treated cells. These data suggest that neither absolute nor relative levels of STAMP uniquely control the growth rates of these ovarian cancer cells. In this case, the effects of decreased intracellular STAMP on cell growth would depend on additional factors in a cell-dependent manner.

The levels of proteins and kinases known to be associated with cancer proliferation and/or apoptosis (p21, p53, ERK, phospho-ERK, AKT, phospho-AKT and cleaved PARP) were examined in the background of STAMP or Lamin silencing to determine if they paralleled the growth rates of the ovarian cancer cells with reduced amounts of STAMP mRNA. No relationship was observed between the expression level of these proteins/kinases on Western blots and the growth rates of A1847, A2780, HeyA8, IGROV1, OVCAR10, PEO-1, and 2008 cells (data not shown). We conclude that changing the intracellular concentration or activation status of any of these proteins does not mediate the effects of STAMP levels on the growth rates of the ovarian tissue culture cells.

### Overexpressed STAMP in stably transfected 293 cells alters properties of GR-mediated transactivation in a cell-specific manner

**Figure 4:**
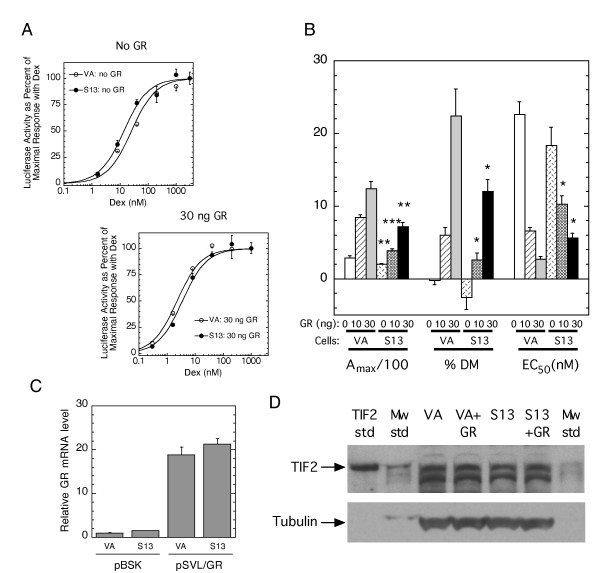
**Effect of elevated STAMP on the properties of GR induction of GREtkLUC reporter gene in 293 cells**. (A) Dose-response curves for GR induction of GREtkLUC reporter with varying amounts of transiently transfected GR plasmid. S13 (containing stably transfected STAMP) and vector control (VA) cells were cotransfected with GREtkLUC reporter and the indicated amounts of GR-encoding plasmid. Dose-response curves were conducted and plotted as described by Tao et al. [32]. (B) Modulation of A_max_, partial agonist activity of the antiglucocorticoid Dex-Mes (%DM), and EC_50 _by elevated STAMP. Four independent experiments with clone S13 and VA cells such as in A, which included assaying the activity of 1 μM Dex-Mes, were analyzed to yield A_max _and EC_50 _in addition to partial agonist activity of 1 μM Dex-Mes (%DM). The average values ± S.E.M. are plotted. * P < 0.05, ** P < 0.005, *** P < 0.0005 for clone S13 vs. the similarly treated clone VA sample. (C) GR mRNA levels in VA and S13 cells. qRT-PCR was used as described in Materials and Methods to determine the level of GR mRNA in VA and S13 cells with or without 30 ng of transiently transfected GR plasmid (± S.E.M. of triplicates). (D) Western blot with TIF2 antibody of cell lysates from cells transfected with no, or 30 ng of GR plasmid. The lower panel shows the equal levels of the loading control, α-tubulin, in each sample.

We speculated that the ability of higher levels of STAMP to produce opposite effects on the growth of 293 and ovarian cancer cells could be due to cell-specific factors. To test this hypothesis, we asked if the same cell-specific differences might also reverse the effects of STAMP on the parameters of GR-regulated gene expression. Previous studies revealed that transiently transfected STAMP in both CV-1 and U2OS.rGR cells not only augment the maximal amount of Dex activity (A_max_), and the partial agonist activity of antisteroids (expressed as percent of maximum Dex activity), but also shift the dose-response curve (and EC_50_) for gene induction to lower concentrations of steroid [15-17]. We therefore asked whether the elevated STAMP levels in 293-derived clone S13 cells would reduce the EC_50 _of the dose-response curve for GR induction of exogenous GREtkLUC reporter relative to the same treatment of vector control VA cells. We used the identical exogenous reporter gene (GREtkLUC) as in our previously published studies [15-17] in order to minimize the differences inherent upon comparing gene induction properties in different cell lines. Due to the relatively low level of GR in 293 cells, we concentrated on the effects of STAMP in the presence of two amounts (10 and 30 ng) of transiently transfected GR plasmid. As expected, the magnitude of change in each parameter was proportional to the amount of transfected GR plasmid [14]. With both amounts of transfected GR, however, the higher level of STAMP in clone S13 cells unexpectedly causes a significant right-shift in the dose-response curve to higher steroid concentrations relative to that seen with vector control VA clone cells (Figs. 4A&B). Similarly, the elevated STAMP in S13 cells decreases both the A_max _and the partial agonist activity of the antiglucocorticoid Dex-mesylate (%DM) (Fig 4B). These changes are statistically significant for each parameter in the presence of both 10 and 30 ng of transfected GR.

A variety of factors could be responsible for the effects of STAMP in Figs. 4A&B being opposite from expected [14]. Two well-known factors that cause increases in A_max_, and partial agonist activity, and decreases in EC_50 _are GR itself and the coactivator TIF2 [14,27,28]. We therefore asked whether higher levels of either GR or TIF2 in VA cells might account for the results of Figs. 4A&B. Unfortunately, the amount of GR protein is too low to detect by Western blotting, even after transient transfection with 30 ng of GR plasmid (data not shown). qRT-PCR was therefore used to determine the GR mRNA levels, which were found to be very similar in the two cell lines both with and without transfected GR plasmid (Fig. 4C). Likewise, Western blotting revealed equal TIF2 protein levels in clone VA and S13 cells both with and without transfected GR (Fig. 4D). Thus, the differences between VA and S13 cells in Figs. 4A&B cannot be explained by VA cells possessing greater amounts of GR and/or TIF2 than S13 cells. We conclude that unidentified cell-specific properties can further alter the effects of changing levels of STAMP on the expression of the same gene(s) in different cells. Therefore, just as was observed for the growth of ovarian cancer cells upon depletion of endogenous STAMP, the other known effects of STAMP are also cell-line dependent.

## Discussion

We report that STAMP affects the physiologically relevant activity of cell growth apparently independent of several steroid hormones. STAMP was initially characterized as a new factor that bound to, and augmented the ability of, the coactivator TIF2 to modulate the transcriptional properties GR, AR, and PR [15-17]. The steroid-independent effects of STAMP on the growth of a variety of cells represent a new activity that differs with the cell line, even within the same cell type. Increased levels of stably transfected STAMP inhibit the growth rate of human embryonic kidney 293 cells while all possible growth responses with changing STAMP are seen in an assortment of ovarian cancer cell lines. We also find a significant correlation between the level of STAMP mRNA and the presence of ovarian cancer. Stage I-III tumor samples possess significantly higher levels of STAMP mRNA.

STAMP was increased in expression in malignant epithelial ovarian cancer compared to normal ovary of unknown epithelial component and the epithelial tumor of low malignant potential. In view of the known heterogeneity of ovarian cancers [24,25], the varied responses to silencing of STAMP in the ovarian cancer cell lines suggest that there may be a select subset of ovarian cancers in which STAMP may regulate behavior. Epithelial ovarian cancer, while initiating in a steroid hormone sensitive organ and known to have functional ER and PR, has not been shown to be steroid hormone driven as in prostate and breast cancer. The lack of response to steroid hormones found herein may be an early signal as to a new role for STAMP that may ultimately explain the altered growth with modulation of STAMP in ovarian cancers. This hypothesis, beyond the scope of the current work, warrants further investigation.

Studies with eleven tissue culture cell lines of ovarian cancer revealed a cell line-dependent effect of reduced STAMP mRNA, with increased or decreased growth rates seen in many lines and no changes in others. Identical cell-specific responses are seen in Fig. 4 regarding another activity of STAMP: its ability to modulate various parameters of GR-regulated gene induction. Thus, the effects of STAMP appear to be sensitive to unknown cell-specific factors. These varying consequences of STAMP are reminiscent of CBP and p300, which can display gene repressive activity [27,29-31] in addition to its more common coactivator properties [10,13]. For STAMP, the different responses are unrelated to the absolute gene expression level of STAMP after knockdown with STAMP siRNA (Fig. 3B). There is also no direct correlation between the growth rate of the cells and the amounts of seven common growth factors and kinases. GEO file searches uncovered several proteins, the expression of which shows some correlation with STAMP on various platforms, but no consistent connection of any protein was evident across the different platforms (data not shown). Thus we have not yet identified the molecular cause for STAMP's effects on cell growth. Nevertheless, given the heterogeneity of ovarian cancers, the statistically significant effect of decreased STAMP mRNA on the growth rates of 5 of 11 ovarian cancer tissue culture cell lines is consistent with an effect that merits further examination.

The steroid-independent effect of STAMP on the growth of stably transfected 293 cells appears to reflect a new activity in addition to its ability to alter the properties of gene induction by GR, PR, and AR [15,16]. The only other characterized domain of STAMP is the tyrosine tubulin ligase-like domain in the amino-terminal third of the molecule, which overlaps the region encoding polyglutamylation activity in some TTL domain proteins including STAMP [18]. We have confirmed that STAMP is able to polyglutamylate tubulin and a few other unidentified proteins (He and Simons, data not shown). While the TTL domain, and polyglutamylation activity, of STAMP are not required for its modulation of GR induction properties [15], they could be relevant for effects on cell growth. It is also possible that steroids will be found to influence the growth properties of the ovarian cancer tissue culture cell lines as their response to different levels of STAMP is often different from that of the 293 cells, where growth is unaffected by steroids. Thus, understanding the possible role of STAMP in ovarian cancer may lead to new insight to the deadly disease.

## Conclusions

This study indicates that one physiological function of the newly described transcriptional cofactor STAMP in several settings is to modify cell growth rates in a manner that can be independent of steroid hormones and may participate in the progression of human cancers. Screening of eight common human cancers revealed that Stage I-III tumor samples of ovarian cancer appear to possess significantly higher levels of STAMP mRNA. Studies with eleven tissue culture cell lines of ovarian cancer uncovered a cell line-dependent effect of reduced STAMP mRNA on cell growth rates. In view of the heterogeneity of ovarian tumors coupled with the dearth of diagnostic tests for ovarian cancers, the current observations suggest that STAMP levels may be of diagnostic value in ovarian cancers and could influence the progression of a sub-set of ovarian tumors.

## Competing interests

The authors declare that they have no competing interests.

## Authors' contributions

YH participated in conceiving the study, designing the experiments, performing the studies with human cancer panels and cells, and revising the manuscript. JAB conducted the experiments in HEK293 cells and helped with manuscript revisions. ECK contributed in the design of the experiments and the revision of the manuscript, SSS participated in conceiving the study, designing the experiments, revising the manuscript, and wrote the initial draft of the paper. All authors read and approved the final manuscript.

## Pre-publication history

The pre-publication history for this paper can be accessed here:

http://www.biomedcentral.com/1471-2407/10/128/prepub

## Supplementary Material

Additional file 1 **STAMP mRNA levels in panel of 8 common human cancers**. The characterization of each sample in a commercially available panel of 8 common human cancers (Origene) is listed. Also given are the Ct values from the qRT-PCR assays and the calculation of the abundance of STAMP mRNA in each sample, relative to the first sample (A1), which was arbitrarily chosen. Click here for file

Additional file 2 **STAMP mRNA levels in panels human ovarian cancers**. The characterization of each sample in two different commercially available panels of human ovarian cancer (Origene; total samples = 96) is presented. Also given are the Ct values from the qRT-PCR assays, the average Ct value (and S.D.) of all samples in each Stage, and the p value of the average STAMP Ct value of each Stage vs. the Stage 0 value. Click here for file
