# Supplementary material for: STAMP alters the growth of transformed and ovarian cancer cells
Source: BMC Cancer. 2010 Apr 7;10:128. doi: 10.1186/1471-2407-10-128 (PMC2858746; doi:10.1186/1471-2407-10-128)
Supplement: Additional file 1 — STAMP mRNA levels in panel of 8 common human cancers. The characterization of each sample in a commercially available panel of 8 common human cancers (Origene) is listed. Also given are the Ct values from the qRT-PCR assays and the calculation of the abundance of STAMP mRNA in each sample, relative to the first sample (A1), which was arbitrarily chosen. [file 1471-2407-10-128-S1.DOC]

|  |  |  |  |  | DATA FOR PANEL OF 8 COMMON HUMAN TUMORS |  |  |  |  |  |
| --- | --- | --- | --- | --- | --- | --- | --- | --- | --- | --- |
|  |  |  |  |  |  |  |  |  |  |  |
| Well position | Gender | Age | Tissue | Appearance | Diagnosis | Tumor grade | Stage | STAMP Ct | ∆Ct re A1 | % re A1 |
| A1 | Female | 44 | Breast / Breast | Normal | Adenocarcinoma of breast, ductal | Adenocarcinoma of breast, ductal | 0 | 33.981 | 0.000 | 100.0 |
| A2 | Female | 45 | Breast / Breast | Normal | Adenocarcinoma of breast, ductal | Adenocarcinoma of breast, ductal | 0 | 33.560 | -0.421 | 133.9 |
| A3 | Female | 32 | Breast / Breast | Normal | Adenocarcinoma of breast, ductal | Adenocarcinoma of breast, ductal | 0 | 34.441 | 0.460 | 72.7 |
| A4 | Female | 47 | Breast / Breast | Tumor | Adenocarcinoma of breast, ductal | Adenocarcinoma of breast, ductal | I | 32.790 | -1.192 | 228.4 |
| A5 | Female | 52 | Breast / Breast | Tumor | Adenocarcinoma of breast, ductal | Adenocarcinoma of breast, ductal | I | 33.972 | -0.010 | 100.7 |
| A6 | Female | 57 | Breast / Breast | Tumor | Adenocarcinoma of breast, ductal | Adenocarcinoma of breast, ductal | IIA | 32.939 | -1.042 | 205.9 |
| A7 | Female | 42 | Breast / Breast | Tumor | Adenocarcinoma of breast, ductal | Adenocarcinoma of breast, ductal | IIA | 33.520 | -0.461 | 137.7 |
| A8 | Female | 63 | Breast / Breast | Tumor | Adenocarcinoma of breast, ductal | Adenocarcinoma of breast, ductal | IIIA | 33.092 | -0.889 | 185.2 |
| A9 | Female | 61 | Breast, right lateral / Breast, right lateral | Tumor | Adenocarcinoma of breast, ductal, lobular | Adenocarcinoma of breast, ductal, lobular | IIIA | 33.198 | -0.784 | 172.1 |
| A10 | Female | 55 | Breast / Breast | Tumor | Adenocarcinoma of breast, ductal | Adenocarcinoma of breast, ductal | IIIC | 33.016 | -0.965 | 195.2 |
| A11 | Female | 45 | Breast / Lung | Tumor | Adenocarcinoma of breast, metastatic | Adenocarcinoma of breast, metastatic | IV | 32.914 | -1.067 | 209.5 |
| A12 | Female | 42 | Breast / Liver | Tumor | Adenocarcinoma of breast, metastatic | Adenocarcinoma of breast, metastatic | IV | 32.764 | -1.218 | 232.6 |
| B1 | Male | 61 | Colon: right / Colon: right | Normal | Adenocarcinoma of colon | Within normal limits | 0 | 33.797 | -0.184 | 113.6 |
| B2 | Male | 91 | Colon: sigmoid / Colon: sigmoid | Normal | Adenocarcinoma of colon | Within normal limits | 0 | 34.308 | 0.327 | 79.7 |
| B3 | Female | 37 | Colon: transverse / Colon: transverse | Normal | Adenocarcinoma of colon, mucinous, signet ring cell | Within normal limits | 0 | 34.617 | 0.636 | 64.4 |
| B4 | Female | 93 | Colon: right / Colon: right | Tumor | Adenocarcinoma of colon | Adenocarcinoma of colon | I | 32.594 | -1.387 | 261.6 |
| B5 | Male | 65 | Colon: right / Colon: right | Tumor | Adenocarcinoma of colon | Adenocarcinoma of colon | IIA | 33.940 | -0.041 | 102.9 |
| B6 | Male | 78 | Colon: sigmoid / Colon: sigmoid | Tumor | Adenocarcinoma of colon | Adenocarcinoma of colon | IIA | 33.395 | -0.586 | 150.1 |
| B7 | Male | 66 | Colon: right / Colon: right | Tumor | Adenocarcinoma of colon | Adenocarcinoma of colon | IIA | 34.193 | 0.211 | 86.4 |
| B8 | Male | 86 | Colon / Colon | Tumor | Adenocarcinoma of colon | Adenocarcinoma of colon | III | 33.971 | -0.010 | 100.7 |
| B9 | Female | 42 | Cecum / Cecum | Tumor | Adenocarcinoma of colon | Adenocarcinoma of colon | IIIB | 34.199 | 0.218 | 86.0 |
| B10 | Male | 61 | Colon: sigmoid / Colon: sigmoid | Tumor | Adenocarcinoma of colon | Adenocarcinoma of colon | IIIC | 35.035 | 1.054 | 48.2 |
| B11 | Male | 63 | Colon: rectosigmoid / Colon: rectosigmoid | Tumor | Adenocarcinoma of colon | Adenocarcinoma of colon | IIIC | 33.259 | -0.722 | 165.0 |
| B12 | Female | 51 | Colon / Lung | Tumor | Adenocarcinoma of colon, metastatic | Adenocarcinoma of colon, metastatic | IV | 33.939 | -0.043 | 103.0 |
| C1 | Male | 71 | Kidney / Kidney | Normal | Carcinoma of kidney, renal cell, clear cell | Within normal limits | 0 | 32.789 | -1.192 | 228.5 |
| C2 | Male | 66 | Kidney / Kidney | Normal | Carcinoma of kidney, renal cell, clear cell | Within normal limits | 0 | 32.925 | -1.056 | 208.0 |
| C3 | Female | 54 | Kidney / Kidney | Normal | Schwannoma | Within normal limits | 0 | 32.282 | -1.700 | 324.8 |
| C4 | Male | 52 | Kidney / Kidney | Tumor | Carcinoma of kidney, renal cell, chromophobe | Carcinoma of kidney | I | 32.661 | -1.320 | 249.7 |
| C5 | Female | 55 | Kidney / Kidney | Tumor | Carcinoma of kidney, renal cell, clear cell | Carcinoma of kidney | I | 33.203 | -0.778 | 171.5 |
| C6 | Male | 52 | Kidney / Kidney | Tumor | Carcinoma of kidney, renal cell, chromophobe | Carcinoma of kidney | I | 33.474 | -0.508 | 142.2 |
| C7 | Female | 57 | Kidney / Kidney | Tumor | Carcinoma of kidney, renal cell | Carcinoma of kidney | II | 33.418 | -0.563 | 147.7 |
| C8 | Male | 59 | Kidney / Kidney | Tumor | Carcinoma of kidney, renal cell, clear cell | Carcinoma of kidney | III | 33.553 | -0.428 | 134.5 |
| C9 | Male | 37 | Kidney / Kidney | Tumor | Carcinoma of kidney, renal cell, clear cell, papillary | Carcinoma of kidney | III | 33.843 | -0.138 | 110.0 |
| C10 | Male | 64 | Kidney / Kidney | Tumor | Carcinoma of kidney, renal cell, papillary | Carcinoma of kidney | III | 35.416 | 1.435 | 37.0 |
| C11 | Male | 70 | Kidney / Lung | Tumor | Carcinoma of kidney, renal cell, clear cell, metastatic | Carcinoma of kidney | IV | 34.047 | 0.066 | 95.5 |
| C12 | Male | 51 | Kidney / Kidney | Tumor | Carcinoma of kidney, renal cell, sarcomatoid | Carcinoma of kidney | IV | 34.939 | 0.958 | 51.5 |
| D1 | Male | 81 | Liver / Liver | Normal | Carcinoma of liver, hepatocellular | Within normal limits | 0 | 33.289 | -0.693 | 161.6 |
| D2 | Male | 86 | Liver / Liver | Normal | Carcinoma of liver, hepatocellular | Within normal limits | 0 | 33.598 | -0.383 | 130.4 |
| D3 | Female | 33 | Liver / Liver | Normal | Nodular hyperplasia of liver, focal | Within normal limits | 0 | 32.917 | -1.064 | 209.1 |
| D4 | Male | 79 | Liver: left lobe / Liver: left lobe | Tumor | Carcinoma of liver, hepatocellular | Carcinoma of liver, hepatocellular | I | 33.879 | -0.102 | 107.3 |
| D5 | Female | 58 | Liver / Liver | Tumor | Carcinoma of liver, hepatocellular | Carcinoma of liver, hepatocellular | I | 33.110 | -0.871 | 182.9 |
| D6 | Male | 66 | Liver / Liver | Tumor | Carcinoma of liver, hepatocellular | Carcinoma of liver, hepatocellular | I | 34.190 | 0.208 | 86.6 |
| D7 | Female | 63 | Liver / Liver | Tumor | Carcinoma of liver, hepatocellular | Carcinoma of liver, hepatocellular | II | 34.425 | 0.443 | 73.5 |
| D8 | Male | 68 | Liver / Liver | Tumor | Carcinoma of liver, hepatocellular | Carcinoma of liver, hepatocellular | II | 33.344 | -0.638 | 155.6 |
| D9 | Female | 62 | Liver: right lobe / Liver: right lobe | Tumor | Carcinoma of liver, hepatocellular | Carcinoma of liver, hepatocellular | II | 33.760 | -0.221 | 116.6 |
| D10 | Male | 71 | Liver / Liver | Tumor | Carcinoma of liver, hepatocellular | Carcinoma of liver, hepatocellular | IIIA | 33.532 | -0.449 | 136.5 |
| D11 | Male | 21 | Liver: left lobe / Liver: left lobe | Tumor | Carcinoma of liver, hepatocellular | Carcinoma of liver, hepatocellular | IV | 34.107 | 0.126 | 91.7 |
| D12 | Male | 66 | Bile duct / Lung | Tumor | Cholangiocarcinoma of liver, metastatic | Cholangiocarcinoma of liver, metastatic | IV | Undetermined | NMF | NMF |
| E1 | Female | 49 | Lung / Lung | Normal | Carcinoma of lung, neuroendocrine | Not Reported | 0 | 33.973 | -0.008 | 100.6 |
| E2 | Male | 79 | Lung / Lung | Normal | Carcinoma of lung, large cell | Not Reported | 0 | 33.114 | -0.867 | 182.4 |
| E3 | Female | 62 | Lung / Lung | Normal | Carcinoma of lung, squamous cell | AJCC G2: Moderately differentiated | 0 | 34.292 | 0.311 | 80.6 |
| E4 | Male | 71 | Lung: left upper lobe / Lung: left upper lobe | Tumor | Carcinoma of lung, squamous cell | AJCC G2: Moderately differentiated | IA | 33.032 | -0.950 | 193.1 |
| E5 | Male | 64 | Lung / Lung | Tumor | Adenocarcinoma of lung | AJCC G3: Poorly differentiated | IB | 31.999 | -1.982 | 395.0 |
| E6 | Female | 85 | Lung / Lung | Tumor | Carcinoma of lung, squamous cell | Not Reported | IB | 33.541 | -0.440 | 135.7 |
| E7 | Male | 72 | Lung / Lung | Tumor | Carcinoma of lung, squamous cell | AJCC G2: Moderately differentiated | IB | 34.366 | 0.385 | 76.6 |
| E8 | Male | 63 | Lung / Lung | Tumor | Carcinoma of lung, non-small cell | AJCC G3: Poorly differentiated | IIB | 33.103 | -0.878 | 183.8 |
| E9 | Male | 71 | Lung / Lung | Tumor | Carcinoma of lung, squamous cell | AJCC G2: Moderately differentiated | IIB | 32.000 | -1.981 | 394.7 |
| E10 | Male | 58 | Lung / Lung | Tumor | Carcinoma of lung, squamous cell | AJCC G3: Poorly differentiated | IIIA | 31.479 | -2.502 | 566.6 |
| E11 | Male | 80 | Lung / Lung | Tumor | Adenocarcinoma of lung | AJCC G3: Poorly differentiated | IIIB | 32.261 | -1.720 | 329.5 |
| E12 | Male | 51 | Lung: right upper lobe / Lung: left upper lobe | Tumor | Carcinoma of lung, non-small cell, metastatic | AJCC G3: Poorly differentiated | IV | Undetermined | NMF | NMF |
| F1 | Female | 70 | Ovary / Ovary | Normal | Within normal limits | Not Reported | 0 | Undetermined | NMF | NMF |
| F2 | Female | 31 | Ovary: right / Ovary: right | Normal | Within normal limits | Not Reported | 0 | 35.680 | 1.699 | 30.8 |
| F3 | Female | 42 | Ovary / Ovary | Normal | Within normal limits | Not Reported | 0 | 34.119 | 0.138 | 90.9 |
| F4 | Female | 29 | Ovary: right / Ovary: right | Tumor | Tumor of ovary, borderline | Not Reported | IA | 32.012 | -1.969 | 391.6 |
| F5 | Female | 43 | Ovary / Ovary | Tumor | Tumor of ovary, borderline | Not Reported | IB | 32.173 | -1.808 | 350.2 |
| F6 | Female | 51 | Ovary: left / Ovary: left | Tumor | Adenocarcinoma of ovary, mucinous | Not Reported | IC | 32.893 | -1.088 | 212.6 |
| F7 | Female | 80 | Ovary: right / Ovary: right | Tumor | Adenocarcinoma of ovary, endometrioid | Not Reported | IIB | 33.519 | -0.463 | 137.8 |
| F8 | Female | 46 | Ovary / Ovary | Tumor | Adenocarcinoma of ovary, endometrioid | Not Reported | IIIA | 33.707 | -0.275 | 121.0 |
| F9 | Female | 52 | Ovary / Ovary | Tumor | Adenocarcinoma of ovary, endometrioid | Not Reported | IIIB | 34.759 | 0.778 | 58.3 |
| F10 | Female | 74 | Ovary: right / Ovary: right | Tumor | Adenocarcinoma of ovary, papillary serous | Not Reported | IIIC | 34.074 | 0.093 | 93.7 |
| F11 | Female | 77 | Ovary: left / Ovary: left | Tumor | Carcinoma of ovary | Not Reported | IIIC | 33.839 | -0.142 | 110.4 |
| F12 | Female | 79 | Ovary / Lymph node | Tumor | Adenocarcinoma of ovary, papillary serous, metastatic | Not Reported | IV | 35.282 | 1.301 | 40.6 |
| G1 | Male | 68 | Prostate / Prostate | Normal | Adenocarcinoma of prostate | 30% glandular epithelium, 70% fibromuscular stroma | 0 | 34.951 | 0.970 | 51.1 |
| G2 | Male | 65 | Prostate / Prostate | Normal | Adenocarcinoma of prostate | 20% epithelium, 80% fibromuscular stroma | 0 | 34.635 | 0.654 | 63.6 |
| G3 | Male | 76 | Prostate / Prostate | Normal | Adenocarcinoma of prostate | 25% epithelium, 70% fibromuscular stroma | 0 | 32.231 | -1.750 | 336.3 |
| G4 | Male | 70 | Prostate / Prostate | Lesion | Carcinoma of bladder, transitional cell | 35% glandular epithelium, 65% stroma | I | 33.203 | -0.778 | 171.5 |
| G5 | Male | 63 | Prostate / Prostate | Lesion | Adenocarcinoma of prostate | 25% epithelium, 75% fibromuscular stroma | II | 33.920 | -0.061 | 104.3 |
| G6 | Male | 70 | Prostate / Prostate | Lesion | Adenocarcinoma of prostate | 45% epithelium, 55% fibromuscular stroma | II | 32.233 | -1.749 | 336.0 |
| G7 | Male | 71 | Prostate / Prostate | Lesion | Adenocarcinoma of prostate | 55% Glandular epithelium, 60% Stroma, 5% Inflammatory cells | II | 32.712 | -1.270 | 241.1 |
| G8 | Male | 56 | Prostate / Prostate | Lesion | Adenocarcinoma of prostate | 65% epithelium, 35% fibromuscular stroma. | II | 33.811 | -0.170 | 112.5 |
| G9 | Male | 63 | Prostate / Prostate | Tumor | Adenocarcinoma of prostate | Inflammation: Mild Lymphoplasmacytic infiltrate | II | 32.990 | -0.992 | 198.8 |
| G10 | Male | 53 | Prostate / Prostate | Lesion | Adenocarcinoma of prostate | 40% glandular epithelium, 60% fibromuscular stroma | III | 32.607 | -1.374 | 259.2 |
| G11 | Male | 65 | Prostate / Prostate | Lesion | Adenocarcinoma of prostate | 55% epithelium, 45% fibromuscular stroma with chronic inflammation. | III | 34.444 | 0.462 | 72.6 |
| G12 | Male | 61 | Prostate / Prostate | Tumor | Adenocarcinoma of prostate | Not Reported | III | 33.803 | -0.178 | 113.1 |
| H1 | Female | 30 | Thyroid gland / Thyroid gland | Normal | Within normal limits | Carcinoma of thyroid, papillary | 0 | 32.970 | -1.012 | 201.6 |
| H2 | Female | 68 | Thyroid gland / Thyroid gland | Normal | Within normal limits | Hamartoma of lung | 0 | 32.928 | -1.053 | 207.5 |
| H3 | Female | 46 | Thyroid gland / Thyroid gland | Normal | Within normal limits | Carcinoma of thyroid, papillary | 0 | 33.378 | -0.603 | 151.9 |
| H4 | Female | 15 | Thyroid gland / Thyroid gland | Tumor | Carcinoma of thyroid, papillary | Carcinoma of thyroid, papillary | I | 34.228 | 0.246 | 84.3 |
| H5 | Female | 28 | Thyroid gland / Thyroid gland | Tumor | Carcinoma of thyroid, papillary | Carcinoma of thyroid, papillary | I | 34.972 | 0.991 | 50.3 |
| H6 | Female | 39 | Thyroid gland / Thyroid gland | Tumor | Carcinoma of thyroid, papillary | Carcinoma of thyroid, papillary | I | 35.059 | 1.077 | 47.4 |
| H7 | Male | 57 | Thyroid gland / Thyroid gland | Tumor | Carcinoma of thyroid, Hurthle cell | Carcinoma of thyroid, Hurthle cell | II | 33.933 | -0.048 | 103.4 |
| H8 | Male | 74 | Thyroid gland / Thyroid gland | Tumor | Carcinoma of thyroid, papillary | Carcinoma of thyroid, papillary | II | 33.544 | -0.437 | 135.4 |
| H9 | Female | 76 | Thyroid gland / Thyroid gland | Tumor | Carcinoma of thyroid, papillary | Carcinoma of thyroid, papillary | III | 34.110 | 0.129 | 91.5 |
| H10 | Female | 52 | Thyroid gland / Thyroid gland | Tumor | Carcinoma of thyroid, follicular | Carcinoma of thyroid, follicular | III | 33.058 | -0.924 | 189.7 |
| H11 | Male | 52 | Thyroid gland / Neck | Tumor | Carcinoma of thyroid, medullary, metastatic | Carcinoma of thyroid, medullary | IVA | 34.396 | 0.415 | 75.0 |
| H12 | Female | 45 | Thyroid gland / Thyroid gland | Tumor | Carcinoma of thyroid, papillary | Carcinoma of thyroid, papillary | IVA | 35.104 | 1.123 | 45.9 |
