# Supplementary material for: STAMP alters the growth of transformed and ovarian cancer cells
Source: BMC Cancer. 2010 Apr 7;10:128. doi: 10.1186/1471-2407-10-128 (PMC2858746; doi:10.1186/1471-2407-10-128)
Supplement: Additional file 2 — STAMP mRNA levels in panels human ovarian cancers. The characterization of each sample in two different commercially available panels of human ovarian cancer (Origene; total samples = 96) is presented. Also given are the Ct values from the qRT-PCR assays, the average Ct value (and S.D.) of all samples in each Stage, and the p value of the average STAMP Ct value of each Stage vs. the Stage 0 value. [file 1471-2407-10-128-S2.DOC]

|  |  |  |  |  | COMBINED DATA OF BOTH ORIGENE OVARIAN CANCER PANELS |  |  |  |  |  |  |  |
| --- | --- | --- | --- | --- | --- | --- | --- | --- | --- | --- | --- | --- |
|  |  |  |  |  |  |  |  |  |  |  |  |  |
| Well position | Cancer | Patient | Tissue | Appearance | Diagnosis | Tumor grade | Stage | Reason samples discarded by authors | STAMP Ct | Ave Ct of Stage | SD | P of Stage vs Stage 0 |
| C01 | ovarian1 | Female-70 | Ovary / Ovary | Normal | Adenocarcinoma of endometrium, papillary serous |  | 0 | Unrelated to ovarian cancer | [32.977] |  |  |  |
| C02 | ovarian1 | Female-37 | Ovary / Ovary | Normal | Carcinoma of cervix, squamous cell |  | 0 | Unrelated to ovarian cancer | [32.083] |  |  |  |
| C03 | ovarian1 | Female-23 | Ovary: left / Ovary: left | Normal | Abscess of tissue |  | 0 |  | 33.602 |  |  |  |
| C04 | ovarian1 | Female-42 | Ovary / Ovary | Normal | Endometriosis |  | 0 |  | 33.106 |  |  |  |
| C05 | ovarian1 | Female-31 | Ovary: right / Ovary: right | Normal | Endometriosis |  | 0 |  | 32.841 | 33.09 | 0.64 |  |
| C06 | ovarian1 | Female-31 | Ovary: left / Ovary: left | Normal | Endometriosis |  | 0 |  | 34.726 |  |  |  |
| C07 | ovarian1 | Female-42 | Ovary / Ovary | Normal | Endometriosis |  | 0 |  | 33.647 |  |  |  |
| C01 | ovarian2 | Female-46 | Ovary: left / Ovary: left | Normal | Leiomyoma of myometrium | Not Applicable | 0 |  | 32.576 |  |  |  |
| C02 | ovarian2 | Female-49 | Ovary / Ovary | Normal | Leiomyoma of myometrium | Not Applicable | 0 |  | 32.858 |  |  |  |
| C03 | ovarian2 | Female-46 | Ovary: right / Ovary: right | Normal | Leiomyoma of myometrium | Not Applicable | 0 |  | 32.765 |  |  |  |
| C04 | ovarian2 | Female-42 | Ovary / Ovary | Normal | Endometrium, secretory | Not Applicable | 0 |  | 32.192 |  |  |  |
| C05 | ovarian2 | Female-33 | Ovary / Ovary | Normal | No residual malignancy | Not Applicable | 0 |  | 32.568 |  |  |  |
| C06 | ovarian2 | Female-40 | Ovary: left / Ovary: left | Normal | Cyst of ovary, follicular | Not Applicable | 0 |  | 32.917 |  |  |  |
| C07 | ovarian2 | Female-45 | Ovary / Ovary | Normal | Endometriosis | Not Applicable | 0 |  | 33.047 |  |  |  |
| C08 | ovarian2 | Female-35 | Ovary: left / Ovary: left | Normal | Endometriosis | Not Applicable | 0 |  | 33.375 |  |  |  |
| C10 | ovarian1 | Female-46 | Ovary: right / Ovary: right | Tumor | Tumor of ovary, papillary serous, borderline | AJCC GB: Borderline malignancy | IA |  | 34.360 |  |  |  |
| C11 | ovarian1 | Female-19 | Ovary: right / Ovary: right | Tumor | Tumor of ovary, papillary serous, borderline | AJCC GB: Borderline malignancy | IA |  | 32.882 |  |  |  |
| D01 | ovarian1 | Female-42 | Ovary: left / Ovary: left | Tumor | Tumor of ovary, serous, borderline | AJCC GB: Borderline malignancy | IA | low malignant, not cancer, not normal | [33.256] | 33.00 | 1.30 | 0.86 |
| D02 | ovarian1 | Female-29 | Ovary: right / Ovary: right | Tumor | Tumor of ovary, borderline | AJCC GB: Borderline malignancy | IA | low malignant, not cancer, not normal | [33.195] |  |  |  |
| D03 | ovarian1 | Female-30 | Ovary: left / Ovary: left | Tumor | Tumor of ovary, mucinous, borderline | AJCC GB: Borderline malignancy | IA |  | 31.770 |  |  |  |
| D07 | ovarian1 | Female-56 | Ovary: right / Ovary: right | Tumor | Tumor of ovary, mucinous, borderline | AJCC GB: Borderline malignancy | IC | low malignant, not cancer, not normal | [31.706] |  |  |  |
| D08 | ovarian1 | Female-74 | Ovary / Ovary | Tumor | Tumor of ovary, serous, borderline | AJCC GB: Borderline malignancy | IC | low malignant, not cancer, not normal | [32.461] |  |  |  |
| D09 | ovarian1 | Female-45 | Ovary / Ovary | Tumor | Tumor of ovary, serous, borderline | AJCC GB: Borderline malignancy | IC | low malignant, not cancer, not normal | [32.272] |  |  |  |
| C08 | ovarian1 | Female-34 | Ovary: left / Ovary: left | Tumor | Carcinoma of ovary, endometrioid | FIGO G2: Moderately differentiated | I |  | 32.136 |  |  |  |
| C09 | ovarian2 | Female-70 | Ovary: left / Ovary: left | Tumor | Carcinoma of ovary, papillary serous | FIGO G3: Poorly differentiated | IA |  | 31.965 |  |  |  |
| C09 | ovarian1 | Female-74 | Ovary / Ovary | Tumor | Adenocarcinoma of ovary, papillary serous | FIGO G2: Moderately differentiated | IA |  | 32.325 |  |  |  |
| C12 | ovarian1 | Female-49 | Ovary: right / Ovary: right | Tumor | Carcinoma of ovary, endometrioid | FIGO G1: Well differentiated | IA | low malignant, not cancer, not normal | [31.473] |  |  |  |
| C10 | ovarian2 | Female-68 | Ovary / Ovary | Tumor | Adenocarcinoma of ovary, papillary serous | FIGO G2: Moderately differentiated | IA |  | 31.237 |  |  |  |
| C11 | ovarian2 | Female-48 | Ovary: left / Ovary: left | Tumor | Adenocarcinoma of ovary, clear cell | Not Reported | IA |  | 32.649 | 31.98 | 0.47 | <0.0001 |
| D04 | ovarian1 | Female-63 | Ovary: right / Ovary: right | Tumor | Adenocarcinoma of ovary, mucinous | FIGO G3: Poorly differentiated | IB | low malignant, not cancer, not normal | [32.842] |  |  |  |
| D05 | ovarian1 | Female-52 | Ovary / Ovary | Tumor | Adenocarcinoma of ovary, endometrioid | FIGO G3: Poorly differentiated | IB | low malignant, not cancer, not normal | [32.360] |  |  |  |
| D06 | ovarian1 | Female-43 | Ovary / Ovary | Tumor | Tumor of ovary, borderline | Not Reported | IB | low malignant, not cancer, not normal | [32.820] |  |  |  |
| C12 | ovarian2 | Female-58 | Ovary: left / Ovary: left | Tumor | Adenocarcinoma of ovary, endometrioid | FIGO G1: Well differentiated | IB |  | 32.320 |  |  |  |
| D01 | ovarian2 | Female-55 | Ovary: right / Ovary: right | Tumor | Adenocarcinoma of ovary, endometrioid | FIGO G3: Poorly differentiated | IB |  | 32.544 |  |  |  |
| D02 | ovarian2 | Female-74 | Ovary: bilateral / Ovary: right | Tumor | Adenocarcinoma of ovary, endometrioid, papillary serous | FIGO G3: Poorly differentiated | IB |  | 31.502 |  |  |  |
| D10 | ovarian1 | Female-51 | Ovary: left / Ovary: left | Tumor | Adenocarcinoma of ovary, mucinous | FIGO G2: Moderately differentiated | IC |  | 31.800 |  |  |  |
| D11 | ovarian1 | Female-58 | Ovary: right / Ovary: right | Tumor | Adenocarcinoma of ovary, endometrioid, squamous features | FIGO G2: Moderately differentiated | IC |  | 32.299 |  |  |  |
| D03 | ovarian2 | Female-65 | Ovary: bilateral / Ovary | Tumor | Carcinoma of ovary, papillary serous | FIGO G3: Poorly differentiated | IC |  | 31.505 |  |  |  |
| D04 | ovarian2 | Female-58 | Ovary / Ovary | Tumor | Carcinoma of ovary, endometrioid | FIGO G2: Moderately differentiated | IC |  | 31.454 |  |  |  |
| D05 | ovarian2 | Female-78 | Ovary: bilateral / Ovary: right | Tumor | Adenocarcinoma of ovary, papillary serous | FIGO G2: Moderately differentiated | IIA |  | 31.184 |  |  |  |
| D06 | ovarian2 | Female-44 | Ovary: bilateral / Ovary: right | Tumor | Carcinoma of ovary, papillary serous | FIGO G2: Moderately differentiated | IIA |  | 32.803 |  |  |  |
| D07 | ovarian2 | Female-73 | Ovary: left / Ovary: left | Tumor | Adenocarcinoma of ovary | FIGO G3: Poorly differentiated | IIA |  | 31.732 |  |  |  |
| D12 | ovarian1 | Female-65 | Ovary / Ovary | Tumor | Adenocarcinoma of ovary, serous | FIGO G3: Poorly differentiated | IIB |  | 31.972 |  |  |  |
| E01 | ovarian1 | Female-80 | Ovary: right / Ovary: right | Tumor | Adenocarcinoma of ovary, endometrioid | FIGO G3: Poorly differentiated | IIB |  | 34.024 | 32.05 | 0.84 | 0.0020 |
| D08 | ovarian2 | Female-67 | Ovary: right / Ovary: right | Tumor | Adenocarcinoma of ovary, serous | FIGO G2: Moderately differentiated | IIB |  | 31.380 |  |  |  |
| D09 | ovarian2 | Female-67 | Ovary: right / Ovary: right | Tumor | Adenocarcinoma of ovary, serous | FIGO G2: Moderately differentiated | IIB |  | 32.244 |  |  |  |
| D10 | ovarian2 | Female-58 | Ovary: left / Ovary: left | Tumor | Adenocarcinoma of ovary, endometrioid | FIGO G3: Poorly differentiated | IIB |  | 32.105 |  |  |  |
| D11 | ovarian2 | Female-58 | Ovary: bilateral / Ovary: right | Tumor | Adenocarcinoma of ovary, papillary serous | FIGO G2: Moderately differentiated | IIB |  | 31.401 |  |  |  |
| E02 | ovarian1 | Female-59 | Ovary: right / Ovary: right | Tumor | Adenocarcinoma of ovary, endometrioid | FIGO G1: Well differentiated | IIC |  | 32.942 |  |  |  |
| D12 | ovarian2 | Female-59 | Ovary: bilateral / Ovary: right | Tumor | Adenocarcinoma of ovary, papillary serous | FIGO G3: Poorly differentiated | IIC |  | 31.333 |  |  |  |
| E01 | ovarian2 | Female-63 | Ovary / Ovary | Tumor | Adenocarcinoma of ovary, serous | FIGO G2: Moderately differentiated | IIC |  | 31.496 |  |  |  |
| E03 | ovarian1 | Female-69 | Ovary: right / Ovary: right | Tumor | Adenocarcinoma of ovary, papillary serous | FIGO G2: Moderately differentiated | III |  | 32.504 |  |  |  |
| E04 | ovarian1 | Female-56 | Ovary: right / Ovary: right | Tumor | Adenocarcinoma of ovary, serous | FIGO G3: Poorly differentiated | III |  | 31.203 |  |  |  |
| E02 | ovarian2 | Female-51 | Ovary / Ovary | Tumor | Adenocarcinoma of ovary, serous | FIGO G2: Moderately differentiated | III |  | 31.596 |  |  |  |
| E03 | ovarian2 | Female-73 | Ovary: right / Ovary: right | Tumor | Adenocarcinoma of ovary, serous | FIGO G3: Poorly differentiated | III |  | 32.922 |  |  |  |
| E04 | ovarian2 | Female-91 | Ovary: bilateral / Ovary | Tumor | Adenocarcinoma of ovary, papillary serous | FIGO G3: Poorly differentiated | III |  | 32.734 |  |  |  |
| E05 | ovarian1 | Female-46 | Ovary / Ovary | Tumor | Carcinoma of ovary, endometrioid | FIGO G2: Moderately differentiated | IIIA |  | 33.956 |  |  |  |
| E06 | ovarian1 | Female-38 | Ovary: right / Ovary: right | Tumor | Tumor of ovary, serous, borderline | AJCC GB: Borderline malignancy | IIIA | low malignant, not cancer, not normal | [31.764] |  |  |  |
| E05 | ovarian2 | Female-75 | Ovary / Ovary | Tumor | Adenocarcinoma of ovary, endometrioid | FIGO G3: Poorly differentiated | IIIA |  | 31.774 |  |  |  |
| E06 | ovarian2 | Female-65 | Ovary / Ovary | Tumor | Adenocarcinoma of ovary, serous | FIGO G3: Poorly differentiated | IIIA |  | 31.568 |  |  |  |
| E07 | ovarian2 | Female-55 | Ovary: bilateral / Ovary | Tumor | Adenocarcinoma of ovary, mucinous | FIGO G2: Moderately differentiated | IIIA |  | 32.892 |  |  |  |
| E08 | ovarian2 | Female-66 | Ovary / Ovary | Tumor | Adenocarcinoma of ovary, serous | FIGO G2: Moderately differentiated | IIIA |  | 32.170 |  |  |  |
| E07 | ovarian1 | Female-41 | Ovary: bilateral / Ovary: right | Tumor | Adenocarcinoma of ovary, papillary serous | FIGO G3: Poorly differentiated | IIIB |  | 33.661 |  |  |  |
| E08 | ovarian1 | Female-75 | Ovary: left / Ovary: left | Tumor | Adenocarcinoma of ovary, serous | FIGO G2: Moderately differentiated | IIIB |  | 31.165 |  |  |  |
| E09 | ovarian1 | Female-52 | Ovary / Ovary | Tumor | Adenocarcinoma of ovary, endometrioid | FIGO G3: Poorly differentiated | IIIB |  | 34.222 |  |  |  |
| E10 | ovarian1 | Female-60 | Ovary: right / Ovary: right | Tumor | Adenocarcinoma of ovary, papillary serous | FIGO G2: Moderately differentiated | IIIB |  | 32.058 | 32.13 | 0.90 | 0.0010 |
| E11 | ovarian1 | Female-52 | Ovary: bilateral / Ovary | Tumor | Adenocarcinoma of ovary, papillary serous | FIGO G3: Poorly differentiated | IIIB |  | 31.868 |  |  |  |
| E12 | ovarian1 | Female-77 | Ovary: right / Ovary: right | Tumor | Tumor of ovary, serous, borderline | Not Reported | IIIB | low malignant, not cancer, not normal | [32.568] |  |  |  |
| F01 | ovarian1 | Female-52 | Ovary / Pelvic wall | Tumor | Adenocarcinoma of ovary, metastatic | Not Reported | IIIB |  | 32.668 |  |  |  |
| E09 | ovarian2 | Female-80 | Ovary: right / Ovary: right | Tumor | Adenocarcinoma of ovary, serous | Not Reported | IIIB |  | 31.937 |  |  |  |
| E10 | ovarian2 | Female-64 | Ovary / Ovary | Tumor | Adenocarcinoma of ovary, papillary serous | FIGO G3: Poorly differentiated | IIIB |  | 30.815 |  |  |  |
| E11 | ovarian2 | Female-66 | Ovary: bilateral / Ovary | Tumor | Carcinoma of ovary, papillary serous | FIGO G3: Poorly differentiated | IIIB |  | 32.773 |  |  |  |
| E12 | ovarian2 | Female-52 | Ovary: left / Ovary: left | Tumor | Adenocarcinoma of ovary, papillary serous | FIGO G3: Poorly differentiated | IIIB |  | 30.951 |  |  |  |
| F02 | ovarian1 | Female-86 | Ovary / Ovary | Tumor | Adenocarcinoma of ovary, papillary serous | FIGO G1: Well differentiated | IIIC |  | 30.990 |  |  |  |
| F03 | ovarian1 | Female-74 | Ovary: right / Ovary: right | Tumor | Adenocarcinoma of ovary, papillary serous | FIGO G3: Poorly differentiated | IIIC |  | 32.015 |  |  |  |
| F04 | ovarian1 | Female-77 | Ovary: left / Ovary: left | Tumor | Adenocarcinoma of ovary, papillary serous | FIGO G3: Poorly differentiated | IIIC |  | 32.569 |  |  |  |
| F05 | ovarian1 | Female-44 | Ovary: right / Ovary: right | Tumor | Adenocarcinoma of ovary, papillary serous | FIGO G3: Poorly differentiated | IIIC |  | 31.599 |  |  |  |
| F06 | ovarian1 | Female-62 | Ovary: right / Ovary: right | Tumor | Adenocarcinoma of ovary, papillary serous | FIGO G3: Poorly differentiated | IIIC |  | 31.182 |  |  |  |
| F07 | ovarian1 | Female-77 | Ovary: left / Ovary: left | Tumor | Carcinoma of ovary | FIGO G3: Poorly differentiated | IIIC |  | 33.245 |  |  |  |
| F08 | ovarian1 | Female-45 | Ovary / Omentum | Tumor | Adenocarcinoma of ovary, papillary serous | Not Reported | IIIC |  | 32.996 |  |  |  |
| F09 | ovarian1 | Female-45 | Ovary: right / Omentum | Tumor | Adenocarcinoma of ovary, serous | Not Reported | IIIC |  | 31.588 |  |  |  |
| F01 | ovarian2 | Female-71 | Ovary: right / Ovary: right | Tumor | Adenocarcinoma of ovary, papillary serous | FIGO G3: Poorly differentiated | IIIC |  | 31.629 |  |  |  |
| F02 | ovarian2 | Female-48 | Ovary / Ovary | Tumor | Adenocarcinoma of ovary, papillary serous | FIGO G2: Moderately differentiated | IIIC |  | 32.223 |  |  |  |
| F03 | ovarian2 | Female-50 | Ovary: bilateral / Ovary: right | Tumor | Carcinoma of ovary, papillary serous | FIGO G3: Poorly differentiated | IIIC |  | 32.383 |  |  |  |
| F04 | ovarian2 | Female-58 | Ovary: left / Ovary: left | Tumor | Adenocarcinoma of ovary, papillary serous | FIGO G2: Moderately differentiated | IIIC |  | 32.672 |  |  |  |
| F05 | ovarian2 | Female-53 | Ovary: right / Ovary: left | Tumor | Adenocarcinoma of ovary, serous | FIGO G3: Poorly differentiated | IIIC |  | 31.235 |  |  |  |
| F06 | ovarian2 | Female-66 | Ovary / Ovary | Tumor | Adenocarcinoma of ovary, endometrioid, papillary serous | FIGO G3: Poorly differentiated | IIIC |  | 30.770 |  |  |  |
| F10 | ovarian1 | Female-81 | Ovary / Ovary | Tumor | Adenocarcinoma of ovary, papillary serous | FIGO G2: Moderately differentiated | IV |  | 31.264 |  |  |  |
| F11 | ovarian1 | Female-77 | Ovary / Colon: sigmoid | Tumor | Adenocarcinoma of ovary, metastatic | Not Reported | IV |  | 34.783 |  |  |  |
| F12 | ovarian1 | Female-79 | Ovary / Lymph node | Tumor | Adenocarcinoma of ovary, papillary serous | FIGO G3: Poorly differentiated | IV |  | 34.226 |  |  |  |
| F07 | ovarian2 | Female-45 | Ovary / Peritoneum | Tumor | Adenocarcinoma of ovary, serous, metastatic | Not Reported | IV |  | 32.680 | 32.37 | 1.40 | 0.083* |
| F08 | ovarian2 | Female-80 | Ovary / Ovary | Tumor | Adenocarcinoma of ovary, endometrioid, papillary serous | FIGO G3: Poorly differentiated | IV |  | 31.830 |  |  | *Mann-Whitney |
| F09 | ovarian2 | Female-68 | Ovary: bilateral / Ovary: right | Tumor | Adenocarcinoma of ovary, serous | FIGO G3: Poorly differentiated | IV |  | 32.777 |  |  |  |
| F10 | ovarian2 | Female-61 | Ovary: bilateral / Ovary: left | Tumor | Adenocarcinoma of ovary, serous | FIGO G3: Poorly differentiated | IV |  | 30.935 |  |  |  |
| F11 | ovarian2 | Female-60 | Ovary / Omentum | Tumor | Adenocarcinoma of ovary, papillary serous, metastatic | Not Reported | IV |  | 30.821 |  |  |  |
| F12 | ovarian2 | Female-63 | Ovary / Omentum | Tumor | Adenocarcinoma of ovary, papillary serous | FIGO G3: Poorly differentiated | IV |  | 32.023 |  |  |  |
